# Application of KMnO4-modified fly ash sorbent for ^226^Ra dose evaluation in bottled mineral waters in Slovakia

**DOI:** 10.1007/s00411-025-01162-y

**Published:** 2025-11-13

**Authors:** Veronika Demovics Silliková, Silvia Dulanská, Klára Gebeová, Michal Trnka, Jana Jakubčinová, Ján Pánik

**Affiliations:** 1 Slovak Medical University, Limbová 12, Bratislava, 833 03 Slovakia; 2 Institute of Inorganic Chemistry, Slovak Academy of Sciences, Dúbravská cesta 9, 845 38 Bratislava, Slovakia; 3 Faculty of Medicine, Institute of Medical Physics and Biophysics, Comenius University Bratislava, Sasinkova 2, 813 72 Bratislava, Slovakia; 4 Faculty of Natural Sciences and Informatics, Department of Botany and Genetics, Constantine the Philosopher University in Nitra, Nábrežie Mládeže 91, Nitra, 949 74 Slovakia

**Keywords:** ^226^Ra, Fly ash, KMnO_4_ modification, Annual effective dose

## Abstract

Monitoring of ^226^Ra in drinking water is critical due to its radiotoxicity and potential health risks. This study presents a novel method for ^226^Ra determination in bottled mineral waters using a KMnO_4_-modified fly ash sorbent. The sorbent, prepared by manganese dioxide surface modification, demonstrated significantly enhanced radium sorption capacity. Method validation, including linear regression and equivalence tests, confirmed that the modified fly ash sorbent and commercial MnO_2_-PAN resin yield statistically equivalent results for ^226^Ra determination in mineral waters. Thus, it is concluded that application of the modified sorbent represents a reliable and cost-effective alternative to commercial methods. Application to 20 commercially available Slovak bottled mineral waters revealed ^226^Ra activity concentrations corresponding to annual committed effective doses ranging from 1 to 154 µSv/y. While the average dose (41.8 µSv/y) remained well below national and international safety limits, three samples exceeded the Slovak regulatory threshold of 100 µSv/y. These findings emphasize the need for ongoing monitoring and demonstrate the suitability of the KMnO_4_-modified fly ash sorbent for routine radiological quality control of bottled mineral waters.

## Introduction

Radium-226 (^226^Ra) is a naturally occurring radioactive isotope that belongs to the uranium decay series and is characterized by a physical half-life of approximately 1,600 years. It predominantly originates from the radioactive decay of ^238^U (Carvalho et al. 2014). Due to its relatively high solubility in water, ^226^Ra can migrate from geological formations into groundwater, including mineral and spring waters, thereby posing a potential human health risk through ingestion (Vengosh et al. 2009). Specifically, chronic ingestion of ^226^Ra-contaminated water is associated with significant health effects. Given its chemical similarity to calcium, radium can accumulate in bones, increasing the risk of bone cancer and other radiological diseases (WHO 2022; IAEA 2014). As a result, international regulatory frameworks, such as those issued by the World Health Organization (WHO) and the European Commission, recommend strict control of radionuclide levels in drinking water. According to WHO guidelines, the committed effective dose from 1 year of drinking water consumption should not exceed 0.1 mSv (100 µSv) (WHO 2022).

Mineral water plays an increasingly important role in daily life. Globally, its consumption continues to grow, especially in developed countries where environmental quality is well maintained and consumers demand high-quality drinking water. The importance of mineral water stems from its natural content of beneficial elements such as magnesium, potassium, calcium, and occasionally therapeutic components like iron or iodine. Such waters are often used for dietary or therapeutic purposes. In most countries, specific legislation regulates the quality and labelling of mineral waters. For example, the European Union has established clear criteria distinguishing natural mineral water from ordinary drinking water. According to these standards, natural mineral water must originate from an underground water source that is protected from contamination, retains its original purity, maintains a stable composition within the limits of natural variability, and provides a specific nutritional or physiological benefit due to its mineral content (Kussmaul 2003). The bottled water industry is further monitored and coordinated by the International Bottled Water Association (Chau and Tomaszewska 2019).

Monitoring of ^226^Ra in drinking water sources, particularly in mineral waters with prolonged water-rock interaction, is crucial for assessing potential radiological hazards. Previous studies have demonstrated considerable variability in ^226^Ra concentrations and resulting annual effective doses across different geographic regions. For example, annual committed effective doses as high as 86 µSv were reported due to ingestion of waters bottled in Croatia (Bronzovic and Marovic 2005), while considerably lower annual committed effective doses were found in Taiwan (7.5 µSv; Kuo et al. 1997) and Iran (0.56 µSv; Sohrabi et al. 1999).

The precise determination of ^226^Ra concentrations relies on a range of analytical techniques, including alpha- and gamma-spectrometry and liquid scintillation counting (LSC) (Hou and Roos 2008; Jobbágy et al. 2017), as well as mass-spectrometric methods - particularly ICP-MS (inductively coupled plasma mass spectrometry) following chemical separation and preconcentration (Copia et al. 2015; Verlinde et al. 2019; Yang et al. 2020; Boudias et al. 2022). Alpha spectrometry is valued for its high sensitivity but requires complex sample preparation. Gamma spectrometry offers a non-destructive alternative but generally has higher detection limits. Liquid scintillation counting, when coupled with pre-concentration techniques, provides a sensitive and efficient method for ^226^Ra quantification (Salonen and Hukkanen 1997).

Recent methodological advancements include the use of composite ion exchangers, such as MnO_2_-PAN (Dulanská et al. 2014), and molecular recognition materials like AnaLig^®^ Sr-01 (Dulanská et al. 2015) and AnaLig^®^ Ra-01 (Verlinde et al. 2019), which significantly enhance radium recovery and reduce analysis time. However, analytical challenges persist, especially in samples with high concentrations of interfering ions like calcium and magnesium ions. Despite these technological improvements, an interlaboratory comparison study revealed that up to 35% of laboratories still encountered significant difficulties in accurately quantifying ^226^Ra in mineral waters, primarily due to low activity concentrations and methodological inconsistencies (Spasova et al. 2011).

Silicon dioxide (SiO_2_), both in its crystalline and amorphous forms, is one of the most abundant materials in the Earth’s crust. However, relatively few studies have focused on the adsorption of Ra^2+^ ions onto silica surfaces (Hamid 2019). Regarding the surface characteristics of silica, Emami et al. (2014) demonstrated that surface charge plays a crucial role in the adsorption process, with higher pH values increasing the negative surface charge and thus enhancing cation adsorption. Given the limited data on the efficiency of Ra^2+^ removal by silica and the hypothesis that silica could act as a good adsorbent due to its structural properties, provided the motivation to explore the modification of fly ash by potassium permanganate (KMnO_4_) to enhance radium sorption.

This work presents a KMnO_4_-modified fly-ash sorbent method for ^226^Ra analysis in bottled mineral waters. The primary aim was to achieve robust recovery with analytical equivalence to the reference MnO_2_-PAN procedure while keeping processing time practical; secondarily, a transparent quality-control framework was implemented, and the method was applied to Slovak products to assess committed effective doses against international limits. In Slovakia, the maximum allowable annual committed effective dose from natural radionuclides in drinking water is set at 0.1 mSv, in accordance with Decree No. 91/2018 Coll. (Ministry of Health of the Slovak Republic 2018), as amended by Decree No. 247/2023 Coll. (Ministry of Health of the Slovak Republic 2024), implementing Directive 2013/51/Euratom (European Commission 2013). Furthermore, for ^226^Ra in natural mineral water, the maximum allowable activity concentration is set at 1.90 Bq/L (MZ SR 2024).

In Slovakia, prior assessments of bottled mineral waters were conducted by the Water Research Institute (Bratislava), which quantified natural radionuclides - including ^226^Ra - and derived total ingestion doses for mineral-water consumption (150 L y^− 1^) (Vráková et al. 2005). Furthermore, Ďurecová et al. (2006) analysed Slovak mineral waters for ^226^Ra, ^224^Ra, ^223^Ra and ^228^Ra and calculated committed effective doses for ingestion.

In the present study the possibility of using a fly ash-based sorbent for the determination of ^226^Ra activities in selected Slovak mineral water samples was investigated, the applied analytical method was evaluated, and associated radiological risks by comparing calculated annual committed effective doses with international safety standards were assessed.

## Materials and methods

### Reagents and materials

The modified sorbent was prepared using fly ash (commercially known as MICROSILICA-SIOXID, supplied by OFZ, a.s., Istebné, Slovakia), potassium permanganate (KMnO_4_, Lachema, Czech Republic), and deionized water. A ^133^Ba radiotracer (certified reference solution, Czech Metrology Institute, Prague; certificate No. 9031-OL-656/12) was used as a chemical-yield monitor. The certified activity concentration (with stated expanded uncertainty, k = 2) is traceable to national standards. Working dilutions were prepared gravimetrically and decay-corrected to the mid-count time. Hydrochloric acid (HCl, Slavus s.r.o., Slovak Republic) was used for the elution of ^226^Ra from the column. Barium chloride dihydrate (BaCl_2_·2H_2_O, p.a., Slavus, Bratislava) and ammonium sulfate ((NH_4_)_2_SO_4_, p.a., Slavus, Bratislava) were used for the preparation of samples for alpha spectrometric analysis. To monitor the separation and behaviour of ^226^Ra, ^133^Ba was employed as a radiotracer.

## Use of ^133^Ba as a radiotracer for ^226^Ra separation

In this study, the radiotracer ^133^Ba was used to determine the recovery of ^226^Ra. This approach is well-established in radiochemical analysis, particularly for environmental and aqueous matrices, due to several key advantages offered by ^133^Ba. Firstly, ^133^Ba exhibits a chemical behaviour similar to that of ^226^Ra, as both belong to the group of alkaline earth metals. During the separation process, such as coprecipitation in the form of sulphates (e.g., Ba(Ra)SO_4_), ^133^Ba mimics the behaviour of ^226^Ra, allowing reliable monitoring of the separation efficiency (Andersson et al. 1996). Secondly, ^133^Ba possesses favourable physical properties: with a physical half-life of about 10.5 years and a prominent 356 keV gamma emission, it is readily detected by gamma spectrometry; in the present work, ^133^Ba was quantified on the 356 keV line. Its relatively low specific activity and ease of handling make it a safer alternative to direct ^226^Ra measurements, significantly reducing radiological exposure and contamination risks in the laboratory (IAEA 2017; Kovács et al. 2006). Furthermore, ^133^Ba is compatible with various detection techniques, including gamma spectrometry and liquid scintillation counting (LSC), offering analytical flexibility. Importantly, since ^133^Ba does not emit alpha particles, it does not interfere with alpha spectrometric measurements of ^226^Ra, thus minimizing potential spectral overlaps (IAEA 2014). Based on these advantages, ^133^Ba was selected as a suitable radiotracer radionuclide for the accurate quantification of ^226^Ra separation yields in the present study.

## Fly ash sorbent preparation and modification

Unmodified fly ash, a by-product of coal combustion containing a high proportion of silicon dioxide, alumina, and iron oxides, was utilized as the base material. A more detailed characterization was provided in a previous study (Silliková et al. 2020). A total of 50 g of fly ash was first washed twice with deionized water to remove fine particulate matter that could potentially clog the column. Subsequently, modification was performed according to the method described in previous studies (Šlesarová 2017; Varga 2007; Ohta et al. 2003). The washed fly ash was digested in 0.5 dm^3^ of 0.5 mol/L KMnO_4_ solution for 2 h at 80 °C. After cooling to room temperature, the modified fly ash was filtered, washed with deionized water, and dried at room temperature. This modification process aimed to enhance the sorption capacity of the material for radium and other bivalent cations through the formation of manganese dioxide coatings on the fly ash surface.

## Evaluation of sorbent performance parameters

In the present study, the method developed for the determination of ^226^Ra was applied to 20 different mineral water samples commercially available in Slovakia. These samples represent a variety of mineralization levels, ranging from low-mineralized waters with less than 200 mg/L to highly mineralized waters exceeding 30 000 mg/L. The overall mineralization of the tested samples ranged from approximately 150 mg/L to 31 500 mg/L. This diversity provides a realistic representation of the types of bottled waters consumed by the population. The results, including recovery yields and measured ^226^Ra activity concentrations, are summarized in Table 1. To guide method optimization, variables expected to affect radium retention and recovery were systematically evaluated: sorption kinetics, pH dependence, elution efficiency, competitive-ion interference, and sample-volume capacity. Experimental designs and instrumentation followed previously reported procedures (Silliková et al. 2023), with adjustments described herein. Detailed conditions for each test are provided in the corresponding subsections.

**Table 1:** ^226^Ra in bottled mineral water determined with modified fly ash sorbent, and annual committed effective dose in bottled mineral waters available in Slovakia

Sample (#)	Total mineralization (mg/L)	*R* ± U (%)	A ± U (Bq/L)	H_E_ (μSv/y)
1	1,540	100 ± 9	0.015 ± 0.002	3.07
2	2,713	100 ± 9	0.080 ± 0.008	16.35
3	508	97 ± 9	0.422 ± 0.051	86.26
4	1,800	100 ± 9	0.012 ± 0.002	2.45
5	1,200	98 ± 9	0.321 ± 0.039	65.61
6	1,000	97 ± 8	0.187 ± 0.022	38.22
7	1,000	100 ± 9	0.022 ± 0.003	4.50
8	300	100 ± 9	0.021 ± 0.003	4.29
9	1,400	98 ± 9	0.013 ± 0.002	2.66
10	1,500	97 ± 8	0.112 ± 0.013	22.89
11	1,200	96 ± 8	0.714 ± 0.086	**145.94**
12	1,000	100 ± 9	0.023 ± 0.003	4.70
13	1,690	100 ± 9	0.011 ± 0.001	2.25
14	1,550	97 ± 9	0.121 ± 0.015	24.73
15	2,400–2,750	99 ± 9	0.013 ± 0.002	2.66
16	150–140	90 ± 9	0.122 ± 0.015	24.94
17	1,628	99 ± 9	0.221 ± 0.027	45.17
18	1,820	98 ± 9	0.704 ± 0.084	**143.90**
19	7,482	75 ± 7	0.215 ± 0.026	43.95
20	31,500	52 ± 6	0.751 ± 0.090	**153.50**

Note: Activity concentration (A) of ^226^Ra (Bq/L); H_E_ – annual committed effective dose (µSv·y^− 1^); R – chemical yield (%); U – total uncertainty

## Determination of ²²⁶Ra in bottled mineral water samples

Twenty mineral water products commercially available in Slovakia were purchased in bottles of 1.0, 1.5, or 2.0 L, and a sample volume between 1.0 and 2.0 L was processed for each product. After cooling, a ^133^Ba radiotracer (70 Bq added per sample) was added. The pH of the samples was adjusted to 6–7 using 0.1 mol/L HCl or 0.1 mol/L NaOH. For column loading, the column (Ø 1 cm; 0.5 g sorbent; BV = 1 mL) was operated at 5 mL·min^− 1^, which allowed processing of typical sample volumes (1.0–2.0 L) within approximately 3–7 h. The column was rinsed with 20 mL of deionized water and, subsequently, ^226^Ra was eluted with 20 mL of 6 mol/L HCl at 0.5 mL·min^− 1^. The samples were then prepared for alpha spectrometric analysis using a 576 A ORTEC alpha spectrometer equipped with an ULTRATM AlphaDetector 450 (detection efficiency 0.28). Minimum detectable activity (MDA) was evaluated following Currie’s formalism (Currie 1999). Under the standard conditions employed in the present study, MDA was 0.001 Bq/L.

### Radiometric analysis

Sample preparation for alpha spectrometry followed the procedure described by Maxwell (2006). Briefly, the eluate was evaporated to dryness. Then the residue was dissolved in 10 mL of 2 mol/L HCl. 1.5 g of ammonium sulfate and 7 µg/mL Ba^2+^ carrier was added. The mixture was placed in an ice bath and treated with ultrasound for 15 min. The Ba(Ra)SO_4_ precipitate was then filtered onto a Tuffryn membrane filter. The filter was dried under an infrared lamp and ^133^Ba yield was determined using a HPGe detector. A Gamma ORTEC HPGe 20,109 P detector and Gamma Vision – 32bit software (EG&G ORTEC) were used for ^133^Ba analysis. The ^226^Ra activity was determined by alpha spectrometry using a 576 A alpha spectrometer with an active area of 600 mm^2^. Spectra were processed using Gamma Vision – 32bit software (EG&G ORTEC). Both the HPGe γ-spectrometer and the α-spectrometry system were operated in a ISO/IEC 17,025–accredited laboratory.

## Measurement uncertainty — alpha spectrometry of ^226^Ra

The activity concentration A (Bq/L) was calculated from the net alpha counts of the ^226^Ra peak as (Eq. 1):

1 $$ A= \frac{(\mathrm{N}\mathrm{s}\:-\:\mathrm{N}\mathrm{b})}{({{\upepsilon\:}}_{{\upalpha\:}}\:\times\:\:\mathrm{t}\:\times\:\:\mathrm{R}\:\times\:\:\mathrm{V})}$$

where $\:\mathrm{N}\mathrm{s}$ and $\:\mathrm{N}\mathrm{b}$ are the sample and background counts, t is the counting time (60,000s), $\:{{\upepsilon\:}}_{{\upalpha\:}}\:$= 0.2 is the alpha-counting efficiency of the ORTEC 576 A spectrometer (calibrated with ^241^Am; activity standard certificate No. 1035–SE–40754–21, supplied by the Czech Metrology Institute, Department of Radionuclide Standard Sources, Prague), R is the radiochemical recovery determined via the ^133^Ba tracer on the HPGe detector (356 keV), and V is the processed sample volume.

The combined standard uncertainty uc(A) was evaluated by the law of propagation of uncertainty (Guide to the Expression of Uncertainty in Measurement, ISO/IEC Guide 98 − 3). The following contributions were included: (i) counting statistics of the net ^226^Ra peak (Ns​,Nb​); (ii) uncertainty of the radiochemical recovery R (HPGe full-energy-peak efficiency at 356 keV, certificate of the reference source, and counting statistics of the ^133^Ba peak); (iii) alpha-efficiency $\:{{\upepsilon\:}}_{{\upalpha\:}}\:$ (calibration with ^241^Am); (iv) background stability and region-of-interest placement; and (V) sample volume V. Under routine conditions (~ 0.20 Bq·L⁻¹), the relative standard uncertainty from net-peak counting was typically 1–3%. The uncertainty of the radiochemical recovery (via ^133^Ba on HPGe) was 2–4%.

The alpha counting efficiency calibration with ^241^Am contributed 2–4%. Background and region of interest (ROI) placement contributed ≤ 1%, and the sample-volume term contributed 0.3–0.8%. Alpha-spectrometry energy/efficiency calibrations used ^241^Am, with routine performance checks according to ISO/IEC 17,025 procedures. Alpha counting efficiency ($\:{{\upepsilon\:}}_{{\upalpha\:}}$=20%) was derived from a ^241^Am calibration performed using the same microprecipitation protocol, planchet diameter, and detector–source geometry as for ^226^Ra. Near the minimum detectable activity (MDA) (~ 0.001 Bq/L), the relative standard uncertainty from net-peak counting increased to 30–40%. The alpha-efficiency calibration remained about 2–4%. Background/ROI placement contributed 2–3%, and the sample-volume contribution stayed at 0.3–0.8%. From these components typical samples uc(A) of about 4–6% was pbtaomed; expanded uncertainty U(A) = k·uc(A) with k = 2 was about 8–12%.

- For the determination of the radiochemical recovery R, a dedicated calibration of the HPGe full-energy-peak efficiency at 356.0 keV was performed using a ^133^Ba tracer. At a counting time of 60,000 s and for the 356 keV γ-line with emission probability Iγ = 0.620, the measured full-energy-peak efficiency of the ORTEC HPGe system in the geometry used was about 3.0%. This calibrated efficiency was subsequently used to evaluate R from the net ^133^Ba peak area. Both the HPGe γ-spectrometer and the α-spectrometry system operated within ISO/IEC 17,025 scope. HPGe energy and full-energy-peak-efficiency calibrations used a certified mixed-gamma reference from the Czech Metrology Institute.

## Method validation and statistical analysis

Methodological validation followed standard practice for complex aqueous matrices (mineral waters), focusing on accuracy, precision, linearity, and robustness while minimizing matrix interferences. The performance of the KMnO_4_ – modified fly-ash sorbent was compared against a commercial MnO_2_–PAN resin (TrisKem International) for ^226^Ra. Both methods were applied to paired aliquots of the same samples (*n* = 18). Agreement was evaluated with linear regression (Eq. 2):

2 $$y=\beta_{0} +\beta_{1}x $$

where *y* represents the results from the KMnO_4_ – modified fly-ash method and *x* those from the MnO_2_–PAN method; the null hypothesis tested was (*H*_0_: *β*_0_ = 0, *β*_1_ = 1).

Pearson’s *R*, regression coefficients with 95% confidence intervals, and residual diagnostics (Jarque–Bera for normality, Cook–Weisberg for heteroskedasticity, Wald’s test for autocorrelation) are reported. Differences in means were assessed by a paired t-test (two-sided, α = 0.05), and differences in precision by an *F*-test on variances. Because heteroskedasticity was detected, regression confidence intervals were also verified using heteroskedasticity-robust standard errors. Analyses were performed with QC-EXPERT v5.3 (Trilobyte, Czech Republic).

### Calculation of annual committed effective dose

To assess the radiological impact of ^226^Ra concentrations in mineral water, the annual committed effective dose was calculated based on the ingestion dose coefficient recommended by the International Commission on Radiological Protection (ICRP) and the International Atomic Energy Agency (IAEA). The dose coefficient for ingestion of ^226^Ra by adults is 2.8 · 10^− 7^ Sv/Bq, equivalent to 0.28 µSv/Bq (ICRP 2012). The dose was calculated assuming a daily water intake of 2 L per person, consumed over 365 days per year. The committed effective dose $\:{H}_{E}$ due to ingestion of ^226^Ra from drinking water was calculated using Eq. (3):

3 $$\:{H}_{E}=\:A\times\:F\times\:t\times\:V$$

where *A* is the activity concentration of ^226^Ra (Bq/L), *F* is the ingestion dose coefficient for adults (0.28 µSv/Bq) (ICRP 2012), *V* is the average daily intake of drinking water (2 L/d) and *t* is the exposure time (365 d/y).

It should be noted that the calculation of annual effective doses is based on an assumed daily intake of 2 L of drinking water, following recommendations of the World Health Organisation (WHO) (WHO 2022). This standardized assumption enables meaningful international comparison of dose estimates and highlights the potential impact of local water consumption patterns.

## Results and discussion

As an initial step, sorption experiments were conducted using unmodified fly ash. The testing of Ba^2+^ carrier concentrations revealed that the unmodified sorbent exhibited low sorption capacity. Above a Ba^2+^ concentration of 1.5 mg/L, the sorption capacity sharply declined. Based on previous success with caesium removal via potassium copper hexacyanoferrate modification, it was hypothesized that proper surface modification would similarly enhance radium (and barium) sorption on fly ash. Consequently, fly ash was modified with 0.5 mol/L KMnO_4_, following literature guidance (Šlesarová 2017; Varga 2007; Ohta et al. 2003) based on the exceptional sorptive properties of manganese oxides. After KMnO_4_ modification, the sorption limit improved significantly from 1.5 mg/L to 20 mg/L for Ba^2+^ ions, indicating a substantial enhancement in sorption performance.

### Testing of modified sorbent characteristics

Environmental waters often contain significant concentrations of competing cations that may influence the sorption of ^226^Ra by occupying active sorption sites. According to (Silliková et al. 2023), monovalent ions such as Na^+^ and K^+^ had no significant effect on radium sorption, even at elevated concentrations. However, the sorbent exhibited selectivity for ^226^Ra even in the presence of high concentrations of competing divalent cations such as Ca^2+^ and Mg^2+^ (up to 50 and 70 mmol·dm^− 3^, respectively). The effect of competing cations is critical for practical applications in natural water systems, where ionic competition may otherwise hinder the effective pre-concentration of target radionuclides.

Additionally, its ability to process water volumes up to 30 L using only 1 g of sorbent emphasizes its practical efficiency. In fact, using 1 g of sorbent, quantitative recovery was maintained up to 30 L (95 ± 5%, *n* = 5), while at 35 L it decreased to 78 ± 7%, indicating column breakthrough. The potential to directly process large volumes of water samples without extensive pre-treatment provides a significant reduction in procedural time compared to alternative separation techniques.

These results demonstrate the practical applicability of the KMnO_4_ – modified fly ash sorbent for environmental and radiological monitoring, enabling efficient processing of large water volumes without compromising recovery. The modified sorbent proved to be a reliable, robust, and scalable material for the pre-concentration of ^226^Ra from mineral waters. The optimized conditions derived from these experiments form the basis for its successful application in the analysis of Slovak mineral water samples, contributing to improved monitoring of radiological safety in drinking water sources.

### Method comparison

Results from the KMnO_4_ – modified fly-ash method closely matched those from MnO_2_–PAN (Pearson *R* = 0.999932; R^2^ = 0.9999). Linear regression (*y* = KMnO_4_ modified fly-ash, *x* = MnO₂-PAN; Eq. 2) gave an intercept *β*_0_ = − 0.0013 (95% confidence interval − 0.0033 to 0.0008; *p* = 0.209) and a slope *β*_1_ = 1.020 (95% confidence interval 1.014–1.026; *p* = 4.7 × 10⁻^6^), indicating excellent agreement with only a small (~ 2%) proportional offset. The mean difference (KMnO_4_ modified fly-ash − PAN) was 0.00277 Bq L⁻¹ (paired *t* = 1.94, d*f* = 17, *p* = 0.069), i.e., there was no significant bias at α = 0.05. Precision did not differ between methods (*F* = 1.04, d*f* = 17,17; *p* ≈ 0.94). For reference, the overall activity concentration levels were comparable (MnO_2_–PAN 0.2008 ± 0.0259 Bq L⁻¹ vs. modified fly-ash 0.2036 ± 0.0265 Bq L⁻¹, mean ± standard deviation (SD)).

Residual diagnostics further validated the model’s assumptions: The Jarque–Bera test rejected normality of the residuals (*p* = 0.0020), the Cook–Weisberg test indicated heteroskedasticity (*p* = 1.79 × 10⁻^5^), and Wald’s test showed no significant autocorrelation (*p* = 0.093). Despite these outcomes, the paired-method comparison supported practical agreement between the approaches (paired *t* = 1.94, *p* = 0.069; variance *F* = 1.04, *p* ≈ 0.94; *β*_*1*_ = 1.020, R^2^ = 0.9999).

These results affirm that the modified fly-ash sorbent provides a statistically reliable alternative to the commercial MnO_2_-PAN method for ^226^Ra analysis in mineral water matrices. When benchmarked against the reference MnO_2_–PAN workflow, the KMnO_4_-modified fly-ash sorbent delivers comparable end-to-end chemistry time for 1–2 L samples (CO_2_ removal, column loading at 5 mL·min^− 1^, rinse and elution) while yielding statistically equivalent results. Beyond analytical performance, the main advantages of the present approach are practical and economic: the sorbent can be prepared in-house from inexpensive precursors using routine laboratory equipment, the material cost per sample is low, and the workflow relies on readily available consumables, which reduces dependence on specialized commercial resins.

### Application of the method to real mineral water samples

Based on the measured activity concentrations (Table 1), the estimated annual committed effective doses ranged from 2.25 to 153.50 µSv/y. Three samples (samples 11, 18, and 20) exceeded the Slovak regulatory limit of 100 µSv/y, with calculated doses of 145.94, 143.90, and 153.50 µSv/y, respectively.

To contextualize the radiological significance of these findings, the average effective dose due to ^226^Ra ingestion from mineral waters commonly available to consumers in Slovakia was compared with published data from other countries (Table 2). The calculated mean annual effective dose for Slovakia was 41.9 µSv/y, based on 20 commercially available samples.

**Table 2:** Average annual effective doses (H_E_) from ^226^Ra ingestion from bottled mineral water in various countries

Country	H_E_ (μSv/y)	References	Samples (bottles)
Slovakia	41.9	Present study	20
Brazil	4.3	Godoy et al. (2001)	36
Croatia	86.0	Bronzovic and Marovic (2005)	11
Egypt	8.0*	Higgy (2000)	7
Hungary	4.1*	Baradács et al. (2001)	14
Romania	67	Moldovan et al. (2009)	23
Japan	12.0	Kinahan et al. (2020)	20
Pakistan	4.1	Fatima et al. (2007)	11
Poland	29	Chmielewska et al. (2020)	30
Turkey	75	Tabar and Yakut (2014)	15

* H_E_ values were calculated based on the information provided in the original papers

These results show that the average annual committed effective dose from ^226^Ra in Slovak mineral waters remains well below the national regulatory limit of 100 µSv/y for most of the investigated samples, as established by Decree No. 91/2018 Coll. (Ministry of Health of the Slovak Republic 2018), as amended by Decree No. 45/2024 Coll. (Ministry of Health of the Slovak Republic 2024), in accordance with Directive 2013/51/Euratom (European Commission 2013). The mean annual effective dose of 41.9 µSv/y obtained in the present study is significantly lower than mean annual effective doses reported for Croatia and Turkey, where annual effective doses of 86 µSv/y and 75 µSv/y were reported, respectively. These higher values can be attributed to specific geological and hydrogeological conditions, including uranium- and thorium-rich crystalline formations and geothermal activity, which enhance radium mobilization. In contrast, countries like Brazil, Pakistan, Hungary, Japan and Egypt reported lower annual effective doses (ranging from 4.1 to 12.0 µSv/y), likely due to lower radium concentrations in drinking water or consumption of treated water sources. Average annual effective doses in Romania and Poland (67 and 29 µSv/y, respectively) were closest to the average value obtained in the present study. The annual effective dose in Croatia (86 µSv/y), however, approached the WHO limit, underscoring regional variability even within geographically close areas.

Although the Slovak mean committed effective dose is well below the regulatory limit of 100 µSv·y^− 1^, three individual samples exceeded this limit. Consistent with prior studies (Chmielewska et al. 2020; Călin et al. 2016), no statistically significant correlation between total mineralization (TDS) and ^226^Ra activity were observed in the present study. This finding supports the applicability of the developed method for routine assessment of radiological safety in bottled mineral waters; nevertheless, routine monitoring remains warranted, and method development should prioritize improving recovery for highly mineralized waters to avoid underestimation. Interestingly, the recovery (R) for ^226^Ra in most tested mineral waters exceeded 95%, regardless of the mineralization level (Table 1). This confirms the robustness and selectivity of the KMnO_4_-modified fly ash sorbent. However, one exception was observed in the sample with the highest total mineral content (31,500 mg/L), where the recovery dropped to 52%. This reduction may be attributed to the high ionic strength or matrix effects limiting the availability of sorption sites or altering radium speciation. A similar but less pronounced decline was observed in sample 19 with 7,482 mg/L, where recovery reached 75%.

These findings demonstrate that while the method is generally effective across a broad range of total mineralization levels, extremely high mineral content may reduce the sorption efficiency of ^226^Ra.

Overall, this study confirms the need for regular monitoring of radionuclides in drinking water and supports the adoption of precise, standardized analytical methods to ensure reliable results and protect public health.

## Conclusion

This study demonstrated the applicability of a KMnO_4_-modified fly ash sorbent for the determination of ^226^Ra in commercially available bottled mineral water samples in Slovakia. The method proved to be effective, with recovery yields exceeding 95% for most samples, and showed robustness even in waters with varying levels of total mineralization. Overall, both methods provide similarly fast and robust preconcentration of ^226^Ra; in fact, the proposed method matched the analytical performance of MnO_2_-PAN while offering cost-effective in-house sorbent preparation, making it attractive for routine monitoring laboratories. The average annual committed effective dose due to ^226^Ra ingestion was calculated as 41.9 µSv/y, which is well below both national and international safety limits. However, three samples exceeded the regulatory limit of 100 µSv/y, highlighting the need for continued monitoring of bottled waters, particularly those with high total mineral content. When compared to other countries, the average annual effective dose is relatively low for bottled mineral water from Slovakia, confirming the generally good radiological quality of local mineral waters. These results place Slovakia among countries with moderate to low natural radionuclide levels in drinking waters. The method proposed here offers a reliable, cost-effective, and selective approach for routine monitoring of ^226^Ra in mineral waters and can be successfully implemented in radiological quality control frameworks to support public health protection. It is recommended that such monitoring be integrated into pre-market quality assessment of bottled mineral waters to ensure continued compliance with safety regulations.

## Data Availability

The datasets generated and analyzed during the current study are available from the corresponding author on reasonable request.

## References

[CR1] Andersson M, Hallstadius L, Holmqvist B (1996) Determination of ^226^Ra in water using ^133^Ba as a yield tracer and liquid scintillation counting. Anal Chim Acta 321(1):47–55. 10.1016/0003-2670(96)00243-7

[CR2] Baradács E, Hunyadi I, Dezső Z, Csige I, Szerbin P (2001) ^226^Ra in geothermal and bottled mineral waters of Hungary. Radiat Meas 34:385–390. 10.1016/S1350-4487(01)00191-3

[CR3] Boudias M, Gourgiotis A, Montavon G, Cazala C, Pichon V, Delaunay N (2022) 226Ra and 137Cs determination by inductively coupled plasma mass spectrometry: state of the Art and perspectives including sample pretreatment and separation steps. J Environ Radioact 244–245:106812. 10.1016/j.jenvrad.2022.106812 10.1016/j.jenvrad.2022.106812 35042022

[CR4] Bronzovic M, Marovic G (2005) Age-dependent dose assessment of ^226^Ra from bottled water intake. Health Phys 88:480–485. 10.1097/01.hp.0000154007.12917.88 15824596 10.1097/01.hp.0000154007.12917.88

[CR5] Călin MR, Begy R et al (2016) Observations on the chemistry of a bottled carbonated mineral water. Romanian J Phys 61:1051–1061

[CR6] Carvalho FP, Oliveira JM, Malta M, Lemos ME (2014) Radioanalytical assessment of environmental contamination around non-remediated uranium mining legacy site and radium mobility. J Radioanal Nucl Chem 299:119–125. 10.1007/s10967-013-2734-1

[CR7] Chau ND, Tomaszewska B (2019) Mineral and bottled water as natural beverages. In: Grumezescu AM, Holban AM (eds) Bottled and Packaged Water, Volume 4: The Science of Beverages. Woodhead Publishing. ISBN 978-0-12-815272-0. 10.1016/C2017-0-02378-9

[CR8] Chmielewska I, Chałupnik S, Wysocka M, Smoliński A (2020) Radium measurements in bottled natural mineral-, spring- and medicinal waters from Poland. Water Resour Ind 24:100133. 10.1016/j.wri.2020.100133

[CR9] Copia L, Nisi S, Plastino W, Ciarletti M, Povinec PP (2015) Low-level 226Ra determination in groundwater by SF-ICP-MS: optimization of separation and pre-concentration methods. J Anal Sci Technol 6:22. 10.1186/s40543-015-0062-5

[CR10] Currie LA (1999) Detection and quantification limits: origins and historical overview. Anal Chim Acta 391:127–134. 10.1016/S0003-2670(99)00105-1

[CR11] Dulanská S, Gardoňová V, Šebesta F, Mátel Ľ (2014) A rapid determination of ^226^Ra in water using composite ion exchanger MnO2–PAN. J Radioanal Nucl Chem 303:47–51. 10.1007/s10967-014-3454-x

[CR12] Dulanská S, Gardoňová V, Remenec B, Mátel Ľ, Cabáneková H (2015) Determination of ^226^Ra using molecular recognition technology product AnaLig^®^ Sr-01. J Radioanal Nucl Chem 309:853–857. 10.1007/s10967-015-4661-9

[CR13] Ďurecová A, Ďurec F, Buršová D (2006) Determination of 226Ra, 224Ra, 228Ra in mineral water samples of the Slovak Republic. In: 15th Radiochemical Conference – Booklet of Abstracts and Conference Programme; Mariánské Lázně, Czech Republic, 23–28 Apr 2006. Czech Technical University, Prague, p. 117. ISBN 80-01-03474-7

[CR14] Emami FS, Puddu V, Berry RJ, Varshney V, Patwardhan SV, Perry CC, Heinz H (2014) Prediction of specific biomolecule adsorption on silica surfaces as a function of pH and particle size. Chem Mater 26:5725–5734. 10.1021/cm5026987

[CR15] European Commission (2013) Council Directive 2013/51/Euratom of 22 October 2013 laying down requirements for the protection of the health of the general public with regard to radioactive substances in water intended for human consumption. Official Journal of the European Union. Available at: https://eur-lex.europa.eu/eli/dir/2013/51/oj/eng

[CR16] Fatima I, Zaidi JH, Arif M, Tahir SNA (2007) Measurement of natural radioactivity in bottled drinking water in Pakistan and consequent dose estimates. Radiat Prot Dosim 123(2):234–240. 10.1093/rpd/ncl093 10.1093/rpd/ncl093 16877468

[CR17] Godoy JM, Amaral EC, da Godoy S MLDP (2001) Natural radionuclides in Brazilian mineral water and consequent doses to the population. J Environ Radioact 53:175–182. 10.1016/S0265-931X(00)00123-5 11378938 10.1016/s0265-931x(00)00123-5

[CR18] Hamid H (2019) Mechanisms of Radium Adsorption on Silica and Barite. Doctoral dissertation. University of South Carolina. Available at: https://scholarcommons.sc.edu/etd/5150

[CR19] Higgy RH (2000) ^226^Ra and Natural Uranium in Egyptian Bottled Mineral Waters. Seventh Conference of Nuclear Sciences & Applications 2: 963–967. Cairo, Egypt, February 6–10. Available at: https://inis.iaea.org/records/vwz9f-kkm06/files/32016607.pdf

[CR20] Hou X, Roos P (2008) Critical comparison of radiometric methods for the determination of radium isotopes in environmental samples. Anal Chim Acta 608:105–139. 10.1016/j.aca.2007.12.012 18215644 10.1016/j.aca.2007.12.012

[CR21] IAEA (2014) The Environmental Behaviour of Radium. Technical Report Series No. 476. International Atomic Energy Agency, Vienna Available at: https://www-pub.iaea.org/MTCD/Publications/PDF/trs476web-45482131.pdf

[CR22] IAEA (2017) Rapid Determination of Radium Isotopes in Drinking Water by LSC. Analytical Quality in Nuclear Applications Series No. 39. Available at: https://www-pub.iaea.org/MTCD/Publications/PDF/IAEA-AQ-39_web.pdf

[CR23] ICRP (2012) Compendium of Dose Coefficients based on ICRP Publication 60. ICRP Publication 119. Ann. ICRP 41(Suppl). Available at: https://journals.sagepub.com/doi/pdf/10.1016/j.icrp.2013.05.003 10.1016/j.icrp.2012.06.038 23025851

[CR24] Jobbágy V, Altzitzoglou T, Malo P, Tanner V, Hult M (2017) A brief overview on radon measurements in drinking water. J Environ Radioact 173:18–24. 10.1016/j.jenvrad.2016.09.019 27745714 10.1016/j.jenvrad.2016.09.019

[CR25] Kinahan A, Hosoda M, Kelleher K, Tsujiguchi T, Akata N, Tokonami S, Currivan L, Vintró LL (2020) Assessment of radiation dose from the consumption of bottled drinking water in Japan. Int J Environ Res Public Health 17:4992. 10.3390/ijerph17144992 32664497 10.3390/ijerph17144992 PMC7400529

[CR26] Kovács T, Somlai J, Szeiler G (2006) Use of ^133^Ba as a Radiotracer for ^226^Ra determination in environmental samples. J Radioanal Nucl Chem 270(3):651–655 Available at:

[CR27] Kuo YC, Lai SY, Huang CC, Lin YM (1997) Activity concentrations and population dose from radium-226 in food and drinking water in Taiwan. Appl Radiat Isot 48:1245–1249. 10.1016/S0969-8043(97)00213-3 9418212 10.1016/s0969-8043(97)00213-3

[CR28] Kussmaul H (2003) Mineral water – sources and analysis. In: Caballero B (ed) Encyclopedia of Food Sciences and Nutrition, Second edition. Academic Press. ISBN 978-0-12-227055-0. 10.1016/B0-12-227055-X/00797-52

[CR29] Maxwell SL III (2006) Rapid method for ^226^Ra and ^228^Ra analysis in water samples. J Radioanal Nucl Chem 270(3):651–655. 10.1007/s10967-012-2257-1

[CR31] Ministry of Health of the Slovak Republic (2018) Decree of the Ministry of Health of the Slovak Republic No. 91/2018 Coll. on details of work and work environment factors in relation to the categorization of work in terms of health risks and on the requirements of a proposal for classifying work into categories. Collection of Laws of the Slovak Republic. Available at: https://static.slov-lex.sk/static/SK/ZZ/2018/91/20180401.html

[CR30] Ministry of Health of the Slovak Republic (2024) Decree of the Ministry of Health of the Slovak Republic No. 45/2024 Coll. on the limitation of exposure of the population from drinking water, natural mineral water, and water suitable for the preparation of food for infants. Collection of Laws of the Slovak Republic. Available at: https://www.slov-lex.sk/pravne-predpisy/SK/ZZ/2024/45/

[CR32] Moldovan M, Cosma C, Encian I, Dicu T (2009) Radium-226 concentration in Romanian bottled mineral waters. J Radioanal Nucl Chem 279(2):487–491. 10.1007/s10967-007-7326-0

[CR33] Ohta T, Saito T, Saito J (2003) Collection of radium isotopes in natural waters by Manganese –. Impregnated Acrylic Fiber Radioisotopes 53(1):1–11. 10.3769/radioisotopes.53.1

[CR34] Salonen L, Hukkanen H (1997) Advantages of low-background liquid scintillation alpha-spectrometry and pulse shape analysis in measuring ^222^Rn, uranium and ^226^Ra in groundwater samples. J Radioanal Nucl Chem 226:67–74. 10.1007/BF02063626

[CR35] Silliková V, Dulanská S, Horník M, Jakubčinová J, Mátel Ľ (2020) Impregnated fly ash sorbent for cesium-137 removal from water samples. J Radioanal Nucl Chem 324:1225–1236. 10.1007/s10967-020-07132-6

[CR36] Silliková V, Dulanská S, Jakubčinová J, Burganová A, Kozlíková K (2023) Fly ash sorbent modified with KMnO_4_ for the separation of important radionuclides. J Radioanal Nucl Chem 332:4335–4341

[CR39] Šlesarová L (2017) Concentration of ^226^Ra and ^90^Sr in water using impregnated polyamide fibre. Dissertation thesis, Faculty of natural sciences of Comenius University, Bratislava

[CR37] Sohrabi M, Beitollahi MM, Hafezi S, Asefi M, Bolourchi M (1999) Effective dose to the public from 226Ra in drinking water supplies of Iran. Health Phys 77:150–153. 10.1097/00004032-199908000-00004 12877336 10.1097/00004032-199908000-00004

[CR38] Spasova Y, Wätjen U, Benedik L, Vasile M, Altzitzoglou T, Beyermann M (2011) Evaluation of EC comparison on the determination of ^226^Ra, ^228^Ra, ^234^U and ^238^U in mineral waters. European Commission, Joint Research Centre, Report EUR 24694 EN Available at: https://publications.jrc.ec.europa.eu/repository/bitstream/JRC62731/jrc62731-final%20report%20eur%2024694.pdf

[CR40] Tabar E, Yakut H (2014) Determination of ^226^Ra concentration in bottled mineral water and assessment of effective doses, a survey in Turkey. Int J Radiat Res 12:193–201. Available at: https://ijrr.com/browse.php?a_code=A-10-1-505&slc_lang=en&sid=1

[CR41] TrisKem, International MnO_2_-Pan Resin, Available at: https://www.triskem-international.com/catalog/products/resins-and-accessories/mno2-pan-resin/bl,product,407,0

[CR42] Varga Z (2007) Preparation and characterization of manganese dioxide impregnated resin for radionuclide pre-concentration. Appl Radiat Isot 65:1095–1100. 10.1016/j.apradiso.2007.05.001 17590345 10.1016/j.apradiso.2007.05.001

[CR43] Vengosh A, Hirschfeld D, Vinson D, Dwyer G, Raanan H, Rimawi O, Ganor J (2009) High naturally occurring radioactivity in fossil groundwater from the middle East. Environ Sci Technol 43:1769–1775. 10.1021/es802969r 19368170 10.1021/es802969r

[CR44] Verlinde M, Gorny J, Montavon G, Khalfallah S, Boulet B, Augeray C, Larivière D, Dalencourt C, Gourgiotis A (2019) A new rapid protocol for 226Ra separation and preconcentration in natural water samples using molecular recognition technology for ICP-MS analysis. J Environ Radioact 202:1–7. 10.1016/j.jenvrad.2019.02.003 30771696 10.1016/j.jenvrad.2019.02.003

[CR45] Vráková M, Babaríková F, Belanová A (2005) The radioactivity of bottled mineral waters. In: XXVII. Days of Radiation Protection. Conference Proceedings; Liptovský Ján, Slovakia, 28 Nov–2 Dec 2005. Nuclear Regulatory Authority of the Slovak Republic, Bratislava, p. 264. ISBN 80-88806-53-4

[CR46] WHO (2022) Guidelines for Drinking-water Quality, Fourth Edition incorporating the First and Second Addenda. World Health Organization, Geneva. Available at: https://www.who.int/publications/i/item/9789240045064 35417116

[CR47] Yang G, Zheng J, Tagami K, Uchida S, Zhang J, Wang J, Du J (2020) Simple and sensitive determination of radium-226 in river water by single column-chromatographic separation coupled to SF-ICP-MS analysis in medium resolution mode. J Environ Radioact 220–221:106305. 10.1016/j.jenvrad.2020.106305 10.1016/j.jenvrad.2020.106305 32560892

